# Effect of butylphthalide on new cerebral microbleeds in patients with acute ischemic stroke

**DOI:** 10.1097/MD.0000000000021594

**Published:** 2020-08-07

**Authors:** Yunlong Ding, Zhiqun Gu, Tingting Zhai, Wenjuan Wang, Yanrong Zhang, Can Wei, Yan Liu, Jiali Niu

**Affiliations:** a Department of Neurology, Jingjiang People's Hospital, the Seventh Affiliated Hospital of Yangzhou University, Jiangsu, China; b Department of Clinical Pharmacy, Jingjiang People's Hospital, the Seventh Affiliated Hospital of Yangzhou University, Jiangsu, China.

**Keywords:** acute ischemic stroke, dl-3-N-butylphthalide, new cerebral microbleeds

## Abstract

**Background:**

To evaluate the effect of dl-3-N-butylphthalide (NBP) on new cerebral microbleeds (CMBs) in patients with acute ischemic stroke (AIS).

**Methods:**

We will prospectively enroll patients with AIS admitted to the stroke center of Jingjiang People's Hospital. Qualified participants will be randomly assigned to either the NBP group (NBP injection) or the control group (NBP injection placebo) in a ratio of 1:1. Patients will complete the brain magnetic resonance imaging within 48 hours and 14 days after stroke onset to observe the CMBs through susceptibility weighted imaging, and evaluate whether the use of NBP will affect the new CMBs in AIS patients. SPSS 20.0 will be used for statistical analyses.

**Result:**

We will provide practical and targeted results assessing the safety of NBP for AIS patients, to provide reference for clinical use of NBP.

**Conclusion:**

The stronger evidence about the effect of NBP on new CMBs in AIS patients will be provided for clinicians.

## 1 Introduction

Acute ischemic stroke (AIS) accounts for about 80% of the incidence of stroke, causing serious economic and social burdens.^[1]^ Currently, intravenous recombinant tissue plasminogen activator (rt-PA) in the early stage is the most effective drug treatment for AIS.^[2]^ Several randomized trials have confirmed that intravenous thrombolysis (IVT) within 4.5 hours of stroke onset improve the clinical outcomes of patients at 3 months.^[3–6]^ Therefore, the cerebrovascular diseases guidelines recommend rt-PA as the first-line treatment for AIS patients. For patients with large vessel occlusion stroke, the recanalization rate of IVT is relatively low. A number of studies have confirmed that endovascular therapy can significantly improve the prognosis of patients with large vessel occlusion stroke.^[7–12]^ However, some patients outside the treatment window lose the chance of thrombolysis or endovascular therapy and even in patients receiving IVT or endovascular treatment, there are still a considerable number of patients with neurological deficits that have not been significantly improved, or even cannot benefit from treatment. Therefore, there is still much room for optimization in the treatment of AIS patients, and it is necessary to find effective drugs to improve the prognosis of AIS patients.

Ischemic stroke usually presents with neurological deficits in the acute phase. Neuroprotective drugs reduce ischemic injury and improve the functional prognosis of patients by various means, and can be used as an effective adjunct to the treatment of patients with ischemic stroke. Previous studies indicated that dl-3-N-butylphthalide (NBP) not only improved neurological function, but also contributed to the long-term outcomes.^[13]^ The mechanisms of NBP for ischemic stroke treatment may involve many complex molecular mechanisms, including anti-oxidant, anti-thrombosis, anti-apoptosis, anti-inflammation, protection of mitochondria, and so on.^[14]^

NBP reduces the content of arachidonic acid and inhibit platelet aggregation and adhesion,^[15]^ which is similar to aspirin. Studies indicated that antiplatelet drugs such as aspirin increased the risk of intracranial hemorrhage^[16–18]^ and the incidence and severity of bleeding are related to the type and dosage of drugs. There were 0.9% serious intracranial hemorrhage conversion events in AIS patients treated with aspirin and clopidogrel, which was higher than 0.4% in aspirin alone group.^[18]^ Hemorrhagic transformation can be used to evaluate the safety of drugs, but no research has been conducted on the bleeding risk of NBP on AIS.

Most of the present research on the safety of AIS treatment drugs is based on symptomatic intracranial hemorrhage (sICH), however, the incidence of sICH is relatively low, which affects the sensitivity of the study. Patients with AIS will develop tiny bleeding lesions that cannot be detected by conventional computed tomography (CT) in the acute phase. These new tiny bleeding lesions detected by susceptibility weighted imaging (SWI) are called new cerebral microbleeds (CMBs).^[19]^ Studies have found that CMBs is a risk factor for sICH, and patients with CMBs are more prone to develop sICH. In patients with ischemic stroke, the incidence of new CMBs is 4.4% to 24.5%,^[20–22]^ which is significantly higher than that of sICH. Therefore, the use of new CMBs to evaluate drug safety for AIS has higher sensitivity. Studies confirmed that aspirin increased the risk of new CMBs.^[23,24]^ NBP reduces the content of arachidonic acid and inhibit platelet aggregation and adhesion, which is similar to aspirin. Therefore, NBP may increase new CMBs in AIS patients. However, NBP can reduce the inflammatory response of AIS and promote blood vessel regeneration after infarction and reduce infarct volume, and studies have shown that the inflammation and infarct volume are risk factors for CMBs,^[25,26]^ suggesting that NBP may reduce new CMBs in AIS. Therefore, it is impossible to speculate the effect of NBP on the new CMBs of AIS. Given that CMBs is a risk factor for intracranial hemorrhage, it is necessary to evaluate whether the use of NBP will increase the new CMBs in patients with AIS.

In this study, we will prospectively include patients with AIS, randomly divide them into an observation group (NBP) and a control group (NBP placebo) and evaluate the effect of NBP on new CMBs.

## 2 Methods

### 2.1 Study design

This is a study protocol for a prospective randomized controlled trail that follows SPIRIT 2013 statement: defining standard protocol items for clinical trials and CONSORT 2010 Statement: updated guidelines for reporting parallel group randomized trials.^[27,28]^

**Figure 1:**
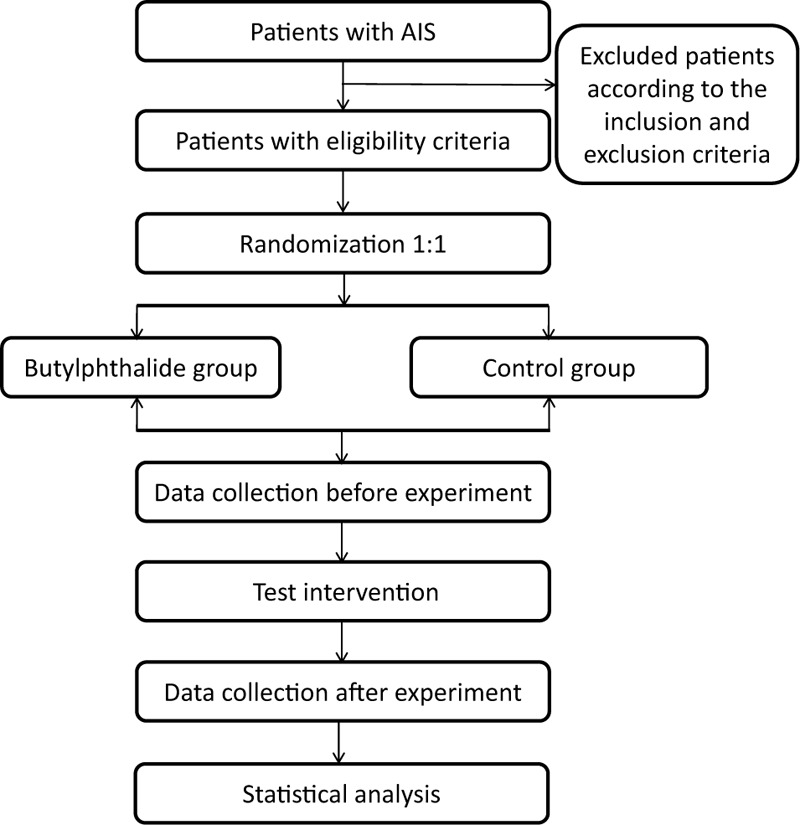
The flowchart of the study protocol.

**Figure 2:**
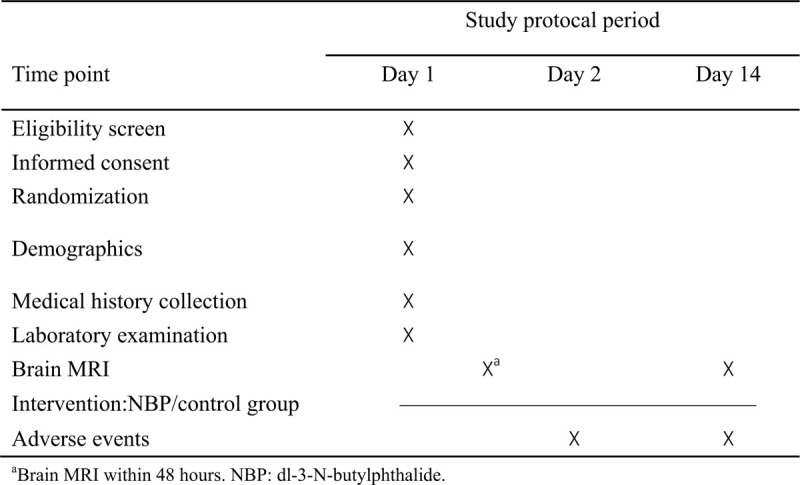
Standard protocol items.

This study will be conducted at the Jingjiang People's Hospital and use new CMBs as indicators to evaluate the safety of NBP in patients with AIS. We will enroll patients with AIS and randomly divided them into the control group and the observation group. The control group will be treated according to the guidelines for AIS and the observation group will be given additional NBP. Patients will complete the brain magnetic resonance imaging (MRI) within 48 hours and 14 days after stroke onset to observe the new CMBs through SWI, and evaluate whether the use of NBP will affect the new CMBs in AIS patients (Figs. 1 and 2). Each participant will be required to sign an informed consent form before enrollment. In addition, they all have the right to withdraw from the study at any time.

### 2.2 Ethics

The study has been approved by the Ethics Committee of Jingjiang People's Hospital (No. 2020-01-006), and it has been registered under the identifier No. ChiCTR2000033486 on the Chinese Clinical Trial Registry on 2020-06-02. Any modifications to the research protocol will be notified to this Human Research Ethics Committee. Informed consent will be obtained from each participant before enrollment. The study bases on the principles of the Declaration of Helsinki and Good Clinical Practice guidelines.

### 2.3 Recruitment

We will prospectively enroll patients with AIS admitted to the stroke center of Jingjiang People's Hospital. The diagnosis of AIS is according to the World Health Organization criteria^[29]^ and confirmed by brain CT or MRI.

### 2.4 Sample size

We used EpiCalc 2000 for sample size estimation, set significance = 0.05, power = 80%, ratio of cases to controls = 1, risk ratio to detect = 2, proportion (%) controls exposed = 15% (4.4%–24.5%). The sample size was 207 in each of the observation group and the control group.

### 2.5 Randomization

Qualified participants will be randomly assigned to either the NBP group or the control group in a ratio of 1:1. Random numbers will be generated by a random number generator in the SPSS version 20.0 software (SPSS, Chicago, IL), which will be operated by a third party who is uninvolved with the treatment and data collection. The drawn letters (A or B) will be placed into opaque envelopes labeled with sequential numbers. The envelopes will be sealed and remain in numerical order in a safe place till the completion of this study. The same researcher (not involved in the study) will prepare the envelopes.

### 2.6 Blinding

In this single-blind study, NBP injection and NBP injection placebo will be produced by CSPC NBP Pharmaceutical Co., Ltd, vensuring that the patients included in the study completely do not know which goods they will receive.

### 2.7 Eligibility criteria

The inclusion criteria are as follows:

(1) aged ≥18 years;
(2) admitted to hospital within 24 hours after stroke onset;
(3) hospitalized with the primary diagnosis of AIS and confirmed by brain CT or MRI and
(4) the patient or their close family member sign an informed consent form.

The exclusion criteria are as follows:

(1) the modified Rankin Scale score >1 before the stroke;
(2) transient ischaemic attack or subarachnoid hemorrhage;
(3) treated with drugs containing NBP before enrollment;
(4) history of coagulopathy, systemic bleeding, thrombocytopenia, or neutropenia;
(5) history of chronic liver disease, liver and kidney dysfunction, increased alanine aminotransferase (more than 3 times the upper limit of normal value), or increased blood creatinine (more than 2 times the upper limit of normal value);
(6) unable to cooperate with 2 MRI examinations;
(7) history of severe cardiopulmonary disease, bradycardia (heart rate below 60 beats/min) or sick sinus syndrome;
(8) life expectancy is less than 14 days or cannot complete the study due to other reasons;
(9) allergic to the drug ingredients in this study; and
(10) other unsuitable situations.

### 2.8 Test drugs

The observation group and control group will be given NBP injection (NBP and sodium chloride injection 100 mL: NBP 25 mg and sodium chloride 0.9 g; CSPC NBP Pharmaceutical Co., Ltd) and NBP injection placebo (NBP sodium chloride placebo 100 mL, NBP 0 mg, sodium chloride 0.9 g, CSPC NBP Pharmaceutical Co., Ltd), respectively.

## 3 Intervention

### 3.1 Treatment

Patients will be grouped immediately after being included in the study. The observation group and the control group will be given NBP injection and NBP injection placebo, respectively, for 14 consecutive days, twice per day. The first injection NBP/NBP placebo should be within 6 hours of stroke onset. In the process of intravenous infusion, a non-PVC infusion set (uniformly equipped by CSPC NBP Pharmaceutical Co., Ltd) must be used. The infusion of NBP time is not less than 50 minutes, and the interval between 2 infusions is not less than 6 hours. If the first infusion start time is after 16:00, then the first day will be given NBP/NBP placebo only once, otherwise 2 times of drugs will be usually given.

We will follow the Chinese guidelines for management of AIS.^[30]^ All patients will receive standards of care, including IVT, endovascular treatment, antiplatelet or anticoagulant therapy based on the patient's condition, nutritional support, and dealing with complications.

Avoid using elixirin, edaravone, or Chinese patent medicine injection containing ginkgo biloba and its extracts during the study. Other medical treatment recommended by the “Chinese Guidelines for the Diagnosis and Treatment of Acute Ischemic Stroke” is permitted.

### 3.2 Brain MRI

The patients will complete the brain MRI within 48 hours and 14 days after stroke onset. By comparing 2 SWI to evaluate the new CMBs, including whether there are new CMBs, the number of CMBs and the location of CMBs.

The patients will be scanned using a SEIMENS MAGNETOM Avanto 1.5 Tesla scanner. Examinations include SWI, diffusion weighted imaging, T1-weighted imaging, T2-weighted imaging, and fluid attenuation inversion recovery. Diffusion weighted imaging scanning sequence: repetition time = 3400 ms; echo time = 102 ms; flip angle = 90; field of view = 230 × 230 mm; acquisition matrix = 192 × 192 mm; no. of excitations = 1.0; thickness = 6.0 mm; gap = 1.2 mm; scan time = 63 seconds. SWI scanning using a 3 dimension- fast field echo (3D-FFE) sequence as follow: repetition time = 17 ms, echo time = 24 ms, flip angle = 15; field of view = 201 × 230 mm; acquisition matrix = 354 × 512 mm; no. of excitations = 1.0; thickness = 1.0 mm; layer = 100; scan time = 209 seconds.

### 3.3 CMBs evaluation

An imaging physician and a neurologist will assess the SWI of the 2 MRIs to evaluate the old CMBs at the time of admission and new CMBs in the acute phase. If the 2 physicians have different opinions on the CMBs judgment, a third senior neurologic physician will intervene until an agreement is reached.

CMBs are examined by SWI. Their diameter are generally less than 5 mm. These small dot-like lesions are thought to be old perivascular collections of hemosiderin deposits. They can be detected by SWI after 48 hours, so SWI examination within 48 hours of stroke onset can identify old CMBs, and SWI at 14 days after onset can identify the new CMBs.

The diagnose of CMBs^[31]^ is as follows:

(1) the low-density signal detected by SWI, round or oval, at least 1/2 of the lesion is surrounded by brain parenchyma, with a clear boundary and a volume of 2 to 10 mm^3^;
(2) T1 and T2 sequences usually do not show abnormal signals;
(3) rule out the diffuse axonal injury caused by brain trauma;
(4) exclude other conditions such as calcification, cavernous hemangioma, and small blood vessel blanking that may show similar signal on SWI.

### 3.4 Outcome assessment

The outcomes will be assessed by independent assessors, who had been trained before participating in this study and blinded to the randomization. All outcome data for participants whether completed or withdrawn during the study will be collected and recorded in the case report form.

Primary outcome: to evaluate the effect of NBP on new CMBs in patients with AIS.

Secondary outcome: to evaluate the effect of NBP on sICH and the National Institute of Health stroke scale score at 14 days of onset.

### 3.5 Adverse events

From the time the patient is enrolled to the last follow-up, any adverse medical events regardless of whether they are causally related to the test drug, will be determined to be adverse events. The researchers will record and report all adverse events. The record includes the occurrence time, severity, duration, measures and outcomes of the adverse events, and separate explanation its potential related drugs.

### 3.6 Quality control and data collection

Due to the fact that any nonstandard or bias input of clinical data can dominate the bias of results, 2 researchers will independently gather the data with case report forms. The collected data will be input into a dedicated computer. The above process will maximize the reliability and safety of the all data. In order to guarantee the quality of the study, all researchers will be required to have an official license for at least 2 years of protocol study and clinical experience.

### 3.7 Data analysis

Statistical analyses will be conducted with SPSS version 20.0 software (SPSS, Chicago, IL). Statistical differences between the 2 groups will be tested using Student *t* test for normally distributed variables or Fisher exact test for dichotomous variables. Two-tailed *P* values < .05 will be considered statistically significant.

## 4 Discussion

NBP is a neuroprotective drug extracted from celery seeds. Its active ingredient is dl-3-NBP, which can increase the levels of nitric oxide and prostacyclin in the brain vascular endothelium, reduce the intracellular calcium ion concentration, inhibit glutamate release, reduce the content of arachidonic acid, inhibit oxygen free radicals, increase the activity of antioxidant enzymes and other mechanisms for multiple pathological links of cerebral ischemic. Ischemic stroke will develop neurological deficits in the acute phase. Neuroprotective drugs can reduce ischemic injury through various ways and improve the functional prognosis of patients. It can be used as an effective auxiliary method for the treatment of patients with ischemic stroke during the acute phase. Our study will evaluate the safety of NBP in AIS patients. If the research proves that NBP can reduce the new CMBs of AIS, it will provide new evidence for the treatment of NBP in AIS; if the research finds that NBP will not affect the new CMBs, it will confirm the safety of NBP; and if the study finds that NBP will increase the new CMBs, it will remind clinicians to carefully evaluate treatment options for patients at greater risk of bleeding.

## Author contributions

Jiali Niu, Yan Liu, Yunlong Ding, Zhiqun Gu, Tingting Zhai, and Wenjuan Wang designed the protocol. The protocol was drafted by Yunlong Ding, Zhiqun Gu, Tingting Zhai, Wenjuan Wang, Yanrong Zhang, and Can Wei. All authors were involved in revising the manuscript critically and gave final approval of the manuscript.
